# Hematological, inflammatory, and hypoxia-responsive adaptations to 18-day normobaric live high-train low training in elite rowers

**DOI:** 10.3389/fphys.2026.1834329

**Published:** 2026-05-13

**Authors:** Anna Kasperska, Hanna Dziewiecka, Wojciech Jankowski, Tomasz Mikulski, Ireneusz Czerniec, Anna Skarpańska-Stejnborn, Joanna Ostapiuk-Karolczuk

**Affiliations:** 1 Department of Biological Sciences, Faculty of Sport Sciences in Gorzów Wielkopolski, Poznań University of Physical Education, Poznań, Poland; 2 Polish Academy of Sciences, Mossakowski Medical Research Institute, Warszawa, Poland

**Keywords:** altitude training, athletes, EPO, hypoxia, inflammation, live high-train low, rowers, VEGF

## Abstract

**Introduction:**

The Live High-Train Low (LH-TL) altitude training method may stimulate erythropoietin (EPO) and vascular endothelial growth factor (VEGF) production, contributing to hematological and physiological adaptations. This study aimed to investigate the effects of an 18-day LH-TL protocol on hematological, inflammatory, and hypoxia-responsive biomarkers in elite rowers.

**Methods:**

Thirteen national-level male rowers were assigned to a hypoxic group (H; n = 8) or a control group (C; n = 5). The H group lived in normobaric hypoxic rooms for 18 days while training in normoxia. Levels of EPO, VEGF, C-reactive protein (CRP), and creatine kinase (CK) were measured along with hematological parameters at baseline, after 6, 12, and 18 days.

**Results:**

EPO levels were higher in the hypoxic group after 18 days compared with the control group. Reticulocyte counts increased after 6 days (15.0 ± 6.2‰) and remained elevated after 18 days, indicating an early erythropoietic response. Hemoglobin showed a non-significant increasing trend, while hematocrit values were significantly higher in the hypoxic group after 18 days. RBC counts remained stable in the hypoxic group, while a slight decline was observed in the control group. VEGF concentrations did not change significantly over time or between groups, although a transient increase was observed around day 12. CRP levels increased after 6 and 18 days, indicating a transient inflammatory response without clinical signs of infection. WBC counts showed a significant group × time interaction, suggesting subtle modulation of immune function in response to LH-TL. CK levels decreased initially but increased again after 18 days, without evidence of excessive muscle damage.

**Conclusion:**

These findings indicate that the LH-TL protocol induces moderate hematological and inflammatory responses in elite rowers. LH-TL may support physiological adaptations relevant to endurance capacity; however, it also represents an additional physiological load that should be carefully monitored during training. Importantly, due to the absence of direct performance measures, the results should be interpreted in terms of physiological adaptations rather than improvements in athletic performance.

**Trial registration ID**: NCT06264193

## Introduction

Hypoxia, defined as a state of reduced oxygen availability, has been extensively investigated in sports science because of its role in driving physiological adaptations. Hypoxic exposure, achieved through natural altitude camps or artificial hypoxic environments, is a legal and widely accepted method for inducing physiological adaptations relevant to endurance capacity in athletes (Neykov et al., 2019; Wilber et al., 2007; Gore et al., 2001; Chang et al., 2023). Several factors related to exercise capacity, including erythropoiesis, aerobic capacity, capillary density, and metabolic efficiency, can be modulated through hypoxic training. Consequently, hypoxic training programs are strategically implemented to enhance physiological adaptations and optimize endurance potential, particularly during preparation for competitions held at sea level.

Altitude training has been associated with a range of muscular and metabolic adaptations, including increased muscle buffering capacity, elevated myoglobin content, and may support capillarization, stimulation of glycolytic enzyme activity, and expansion of mitochondrial volume in skeletal muscle (Sinex and Chapman, 2015; Lemieux and Birot, 2021; Chinapong et al., 2021). Hypoxic conditions can be induced either by natural altitude exposure or simulated environments such as hypoxic tents, chambers, or dedicated altitude rooms (Wilber, 2011; Wilber et al., 2007; Viscor et al., 2018; Kasperska and Zembron-Lacny, 2020). As a result, altitude training remains one of the most widely applied strategies to enhance endurance capacity in elite athletes, particularly when the hypoxic dose and training load are appropriately managed (Wilber et al., 2007; Bonato et al., 2023; Mujika et al., 2019).

At the molecular level, hypoxia activates hypoxia-inducible factors (HIFs), which play a central role in cellular oxygen sensing and regulate the expression of key genes involved in oxygen transport and vascular adaptation, including erythropoietin (EPO) and vascular endothelial growth factor (VEGF) (Semenza, 2022; Cerda-Kohler et al., 2022; Haase, 2013). In skeletal muscle, activation of these pathways enhances vascular function and may also influence inflammatory responses (Ramakrishnan et al., 2014).

EPO is a major regulator of erythropoiesis and stimulates the production of red blood cells and reticulocytes, leading to increased hemoglobin concentration and hematocrit levels. These changes may improve the blood’s oxygen-carrying capacity and may enhance endurance physiological adaptation (Montero et al., 2017; Semenza, 2022; Wehrlin and Hallén, 2006). Previous studies have reported significant increases in circulating EPO following hypoxic exposure, with elevations observed both after prolonged exposure and after relatively short hypoxic stimuli (Sinex and Chapman, 2015; Chapman et al., 2010).

VEGF plays a key role in angiogenesis by stimulating endothelial cell proliferation and capillary growth, which supports oxygen delivery to active skeletal muscles during exercise (Neykov et al., 2019). In contrast to erythropoietic responses, VEGF adaptations to hypoxic training appear to be more variable and less consistent across studies. Wiśniewska et al. (2020) observed no significant changes in VEGF following LH-TL, while other studies suggest that angiogenic responses may depend on hypoxic dose, exposure duration, and individual responsiveness (Wahl et al., 2013; Li et al., 2020).

In the present study, VEGF levels showed a transient increase around day 12 but did not reach statistical significance. This pattern may reflect the dynamic and time-dependent nature of angiogenic signaling during hypoxic exposure, and suggests that longer or more intense hypoxic stimuli may be required to elicit robust VEGF-mediated adaptations. These vascular adaptations may contribute to enhanced oxygen diffusion and tissue perfusion during endurance exercise.

While hypoxia is well known to stimulate erythropoiesis through increased EPO production, emerging evidence suggests that it may also influence systemic inflammation and immune function (Burtscher et al., 2024b). This interaction is particularly relevant in the context of physical training, where recovery and physiological adaptation must be carefully balanced. Hypoxia-induced stabilization of HIF transcription factors can stimulate the production of pro-inflammatory mediators such as C-reactive protein (CRP), tumor necrosis factor-α (TNF-α), and interleukin-6 (IL-6) (Baygutalp et al., 2021). Short-term hypoxic exposure may support adaptive immune responses and tissue remodeling; however, prolonged exposure combined with intense physical activity may exacerbate inflammatory processes and impair recovery (Burtscher et al., 2024a). Markers such as CRP may therefore reflect systemic responses to the combined effects of hypoxic exposure and training load, with transient increases observed following acute training protocols (Baygutalp et al., 2021; Ostapiuk-Karolczuk et al., 2025). Importantly, exercise itself is a well-established stimulus for acute-phase inflammatory responses, with CRP concentrations increasing in relation to exercise intensity and duration (Cerqueira et al., 2020). Similarly, transient elevations in CRP have been reported following exercise without indicating pathological conditions, reflecting physiological stress and recovery processes (Mouridsen et al., 2014).

Among the different altitude training models, the Live High-Train Low (LH-TL) strategy is widely recognized as one of the most effective hypoxic training methods for improving endurance capacity while maintaining high-intensity training in normoxic conditions (Lundby and Robach, 2016; Czuba et al., 2018; Park et al., 2019; Chang et al., 2023). The mechanisms underlying improvements in exercise capacity are primarily related to enhanced erythropoiesis and increased oxygen transport capacity (Khodaee et al., 2016; Semenza, 2022). The effectiveness of LH-TL interventions depends on the applied hypoxic dose, which includes both the altitude level and the duration of daily exposure. An “optimal hypoxic dose” has been proposed to involve exposure to altitudes of approximately 2000–3000 m for at least 12–16 hours per day to induce meaningful hematological adaptations (Wilber et al., 2007). However, the effectiveness of altitude training varies considerably among individuals, and improvements in exercise capacity are not always observed, highlighting the importance of individual responsiveness to hypoxic stimuli (Chen et al., 2023).

Therefore, the present study aimed to investigate the effects of an 18-day Live High-Train Low (LH-TL) protocol using normobaric hypoxic rooms on erythropoietin (EPO), vascular endothelial growth factor (VEGF), and selected hematological parameters in elite rowers. In addition, the study aimed to examine whether hypoxic exposure during the LH-TL intervention may act as a systemic physiological stressor by evaluating markers of inflammation (CRP) and muscle damage (CK). By integrating hematological, molecular, and inflammatory indicators, this study sought to provide a broader understanding of both the adaptive and potential stress-related responses to LH-TL exposure in elite endurance athletes. The study focused on systemic markers of hypoxia adaptation in an applied LH-TL intervention, without direct assessment of intracellular signaling pathways.

## Materials and methods

### Study

**Figure 1:**
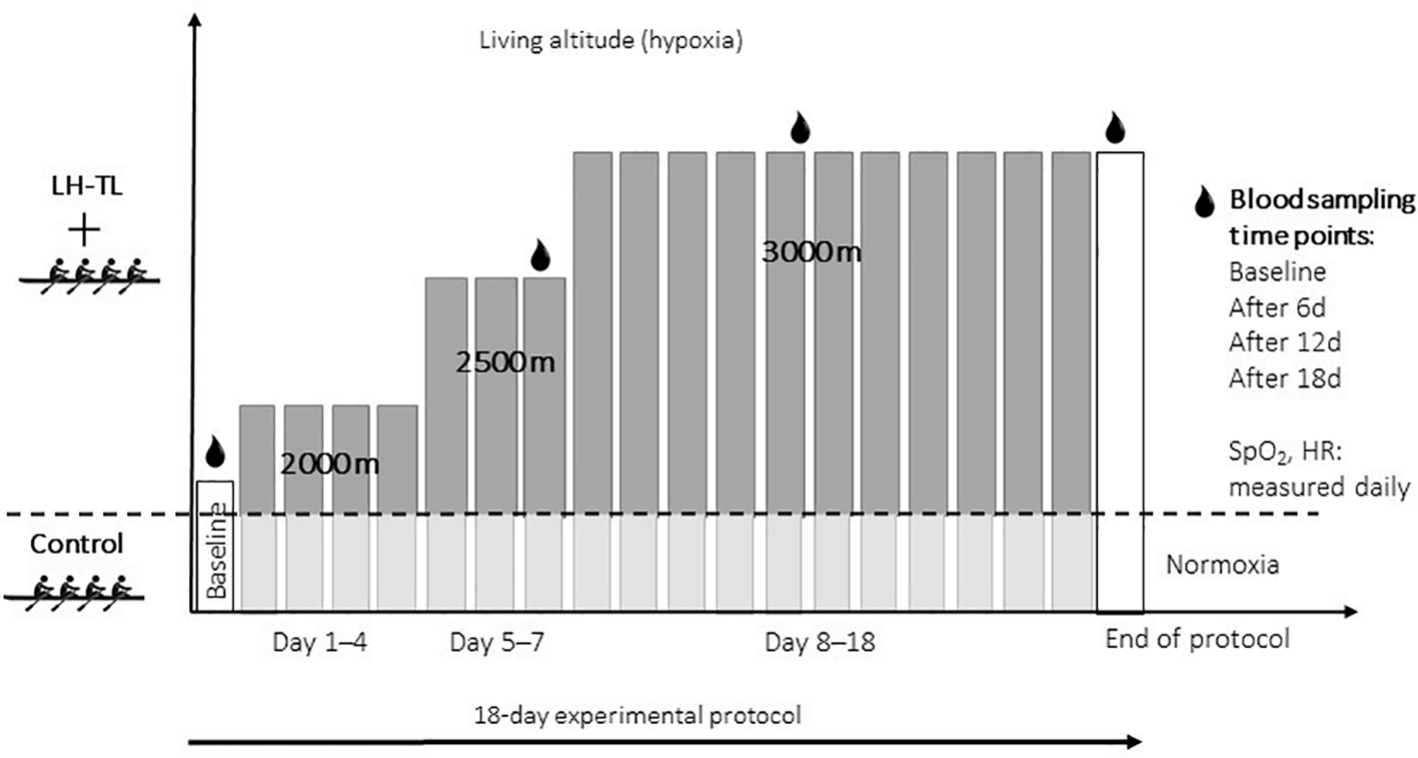
Experimental design of the 18-day live high-train low (LH-TL) protocol. SpO_2_, blood oxygen saturation; HR, heart rate.

This was a parallel-group, prospective, controlled intervention study conducted over an 18-day training camp at the Olympic Sports Center in Poland. The study investigated the physiological effects of a Live High-Train Low (LH-TL) altitude training protocol in elite-level rowers using normobaric hypoxic rooms. The overall study design is illustrated in **Figure 1**.

### Participants

Thirteen elite male rowers from the national team participated in the study (N = 13) (**Table 1**). Participants were allocated to the hypoxic and control groups based on logistical constraints related to the training camp setting, including the limited number of hypoxic rooms, the total number of athletes attending the camp, and accommodation capacity within the Olympic Training Center. This was a parallel-group, prospective, quasi-experimental controlled intervention study conducted over an 18-day training camp at the Olympic Sports Center in Poland.

**Table 1:** Anthropometric characteristics of the study participants (mean ± SD).

Variable	Hypoxic group n = 8	Control group n = 5
Age (years)	26 ± 4	29 ± 5
Height (cm)	197.5 ± 5.3	196 ± 6.0
Weight (kg)	100.8 ± 6.3	94.1 ± 6.2
BMI (kg/m^2^)	25.9 ± 1.6	24.5 ± 1.8
FM%	12.1 ± 3.9	11.1 ± 2.6
FM (kg)	12.3 ± 4.4	10.5 ± 2.8
FFM (kg)	88.5 ± 4.7	83.6 ± 4.7

BMI, body mass index; FM, fat mass; FFM, free fat mass.

1. Hypoxic group (H) (n = 8; age: 26 ± 4 years) - followed the standard training program and were additionally exposed to the LH-TL protocol using normobaric hypoxic rooms.
2. Control group (C) (n = 5; age: 29 ± 5 years) - followed the same training program while living under normoxic conditions.

Within these constraints, group allocation was performed without systematic selection based on capacity or physiological characteristics; however, potential selection bias cannot be fully excluded. Group sizes differed due to the limited availability of hypoxic rooms. The rowers were classified as elite based on their membership on the national team, participation in national and international competitions, advanced physical conditioning, and extensive rowing experience.

Due to the nature of the intervention, which involved prolonged exposure to hypoxic rooms, neither participants nor researchers were blinded to group allocation. All participants who were enrolled in the study were included in the intervention and completed the protocol. No participants were excluded after allocation, and no dropouts occurred during the study. Therefore, all participants were included in the final statistical analysis.

No statistically significant differences between groups were observed for any anthropometric variables at baseline.

The relatively small sample size reflects the limited availability of elite athletes participating in the national team training camp, which is common in studies involving high-training athletes.

Iron status. Baseline iron (Fe) and TIBC (Total Iron Binding Capacity) values were within reference ranges in both groups (H: 26.9 ± 6.5 and 56.1 ± 5.8 µmol/L; Con: 25.0 ± 6.2 and 56.8 ± 4.1 µmol/L; reference ranges: Fe 11-28 µmol/L, TIBC 40.8-76.6 µmol/L), confirming normal iron status before the LH-TL intervention. Analyses were performed in a certified laboratory (Poland, ISO 9001:2008).

### Training protocol

Rowers were observed during a 3-week training camp conducted in the preparatory period, during which 83.1% of the training load consisted of general training and 16.9% of specific rowing training. The weekly training program included ergometer rowing, mountain running, gym-based strength exercises, swimming, team games, general development exercises, and functional muscle training. The total training volume during the camp amounted to 6390 minutes (106 h 30 min) (**Table 2**).

**Table 2:** Weekly rowers’ training schedule during the camp.

Training session character	Weekly training schedule (min/day)							Training components (%)
	Day 1	Day 2	Day 3	Day 4	Day 5	Day 6	Day 7	
Run/walk in the mountains	150	150	150	–	150	–	150	42.3
Rowing ergometer	–	130	–	120	–	120	–	16.9
Swimming	40	–	40	–	40	–	–	5.6
Gym exercises	120	–	120	–	120	–	–	16.9
Functional muscle exercises	–	30	–	30		30	–	8.9
Team games	40	–	40	–	40	120	–	9.4
Biological recovery*	–	–	–	90-120	–	–	90-120	

* hydromassage, brine, sauna, dry massage.

### Intervention (LH-TL protocol)

The LH-TL protocol was carried out over 18 days using normobaric hypoxic rooms equipped with the AirZone^®^ system. Simulated altitude exposure was progressively increased in three stages to allow athletes to acclimatize to increasing altitude: Days 1-4: 2000 m (FiO_2_ ≈ 16.4%); Days 5-7: 2500 m (FiO_2_ ≈ 15.4%); Days 8-18: 3000 m (FiO_2_ ≈ 14.5%). The progressive increase in simulated altitude (2000 m → 2500 m → 3000 m) was applied to facilitate gradual acclimatization and minimize excessive physiological strain. Initial exposure to moderate hypoxia (2000 m) allows early hematological adaptation, while subsequent increases provide a stronger hypoxic stimulus required to elicit more pronounced erythropoietic and molecular responses.

Participants in the hypoxic group lived and slept in hypoxic rooms for at least 16 hours per day. They exited the hypoxic environment only for training sessions and meals. The control group resided at the same camp and followed the same training schedule and diet, but under normoxic conditions. Participants from both groups followed the same biological recovery and standardized diet provided during the training camp. All meals were prepared and served at the same location, ensuring similar energy intake and nutritional composition for both groups.

### Safety and monitoring

The AirZone^®^ system allows precise control of oxygen concentration by modifying the ambient air composition. Room conditions were centrally monitored to ensure the accuracy and stability of hypoxic exposure, and an automatic alarm was triggered if disturbances in air composition occurred. Each room was equipped with an emergency button that allowed athletes to immediately turn off the hypoxic system if they experienced any discomfort or other symptoms. Athletes participating in the LH-TL protocol were continuously monitored for safety by a physician and a nurse. No adverse events were reported during the intervention.

### Measurements

Venous blood samples were collected four times: at baseline (beginning of the camp), and after 6, 12, and 18 days of LH-TL intervention, to capture early, intermediate, and sustained phases of physiological adaptation to hypoxia during the LH-TL intervention. All samples were obtained in the morning after awakening between 07:00 and 07:30 AM following an overnight fast. Blood samples were centrifuged at 3000 rpm for 10 min at 4 °C to obtain serum. The serum was immediately separated and stored at -80 °C until analysis.

Erythropoietin (EPO) and vascular endothelial growth factor (VEGF) concentrations were determined using enzyme-linked immunoassays (ELISA) methods with commercial kits (DRG International Inc., USA; cat. no. EIA-3646, EIA-4819, respectively). High-sensitivity C-reactive protein (hsCRP) was determined using a high-sensitivity immunoenzymatic assay with diagnostic kits (DRG International Inc., USA; cat. no. EIA-3954). The total creatine kinase (CK) activity was determined using a spectrophotometer and a reagent kit (BioMaxima S.A., Poland; cat. no. 1-233-0060). Hematological variables (Hb, Ht, RBC, WBC, and Ret) were determined by a professional accredited laboratory (Poland, ISO 9001:2008).

Peripheral oxygen saturation (SpO_2_) and heart rate (HR) were measured twice daily throughout the LH-TL protocol, at rest in the morning and in the evening. Measurements were performed using a pulse oximeter (Tech-Med, ISO: 13485). The perfusion index (PI) was not assessed, as the study focused on systemic oxygen saturation as the primary outcome.

Before arriving at the training camp, athletes were instructed to rest for 48 hours, avoiding any physical activity.

Anthropometric measurements were taken at the beginning of the training camp. Body composition parameters, including body mass index (BMI), fat mass (FM), and free fat mass (FFM), were estimated using the bioelectrical impedance analysis (BC-418 analyzer, Tanita, Japan).

### Statistical analysis

Data were analyzed using GraphPad Prism 10 (GraphPad Software, Boston, USA). Results are presented as mean ± standard deviation (SD), with statistical significance set at p<0.05. Normality was confirmed using the Shapiro-Wilk test. Differences were assessed using two-way repeated-measures ANOVA to evaluate the effects of time, group, and their interaction (group × time), followed by Tukey’s *post-hoc* test where appropriate. Effect sizes were expressed as partial eta squared (η²p) and interpreted according to conventional thresholds (small: 0.01-0.06; medium: 0.06-0.14; large: >0.14) (Cohen, 1988). Homogeneity of variances was verified using the Brown-Forsythe test, and the Greenhouse-Geisser correction was applied when the assumption of sphericity was violated. Anthropometric variables were analyzed using Welch’s test. Due to the inclusion of elite athletes participating in a centralized training camp, the sample size was determined by participant availability rather than *a priori* power calculation, which is common in high-training sport research. The repeated-measures design increased statistical sensitivity by reducing inter-individual variability; however, the study may have been underpowered to detect small-to-moderate between-group differences. *Post hoc* power analysis indicated low statistical power for several outcomes, including RBC (1-β = 0.22), CRP (1-β = 0.23), and WBC (1-β = 0.25); however, these results should be interpreted with caution, as they are dependent on observed effect sizes and do not provide additional information beyond the reported results.

### Inclusion and exclusion criteria

Inclusion Criteria: male elite-level rowers actively competing as members of the national rowing team, aged between 18 and 35 years; engaged in regular endurance training (≥5 sessions per week) for at least 3 years; in good health as confirmed by a medical doctor; free of any injury, infection or illness limiting training or potentially affecting hematological or inflammatory markers; no residence, training, or exposure to natural or simulated hypoxia (altitude >1500 m) within the past 3 months.

Exclusion Criteria: history of cardiovascular, respiratory, hematological, or metabolic disorders; any acute illness, or infection; use of medications that could affect inflammatory or hematological parameters; known intolerance to hypoxia, or prior adverse reactions to altitude exposure; use of performance-enhancing drugs, iron supplementation within 6 months before the study; participation in another interventional study within 30 days before enrollment.

### Ethics statement

All participants received detailed information about the study and provided written consent before their contribution, in accordance with the Declaration of Helsinki. The study design was approved by the Poznan University of Medical Sciences Ethics Committee (Poland; decision no. 843/14). The study followed CONSORT guidelines and was registered at ClinicalTrials.gov (ID: NCT06264193; registered on 10/01/2024).

## Results

**Figure 2:**
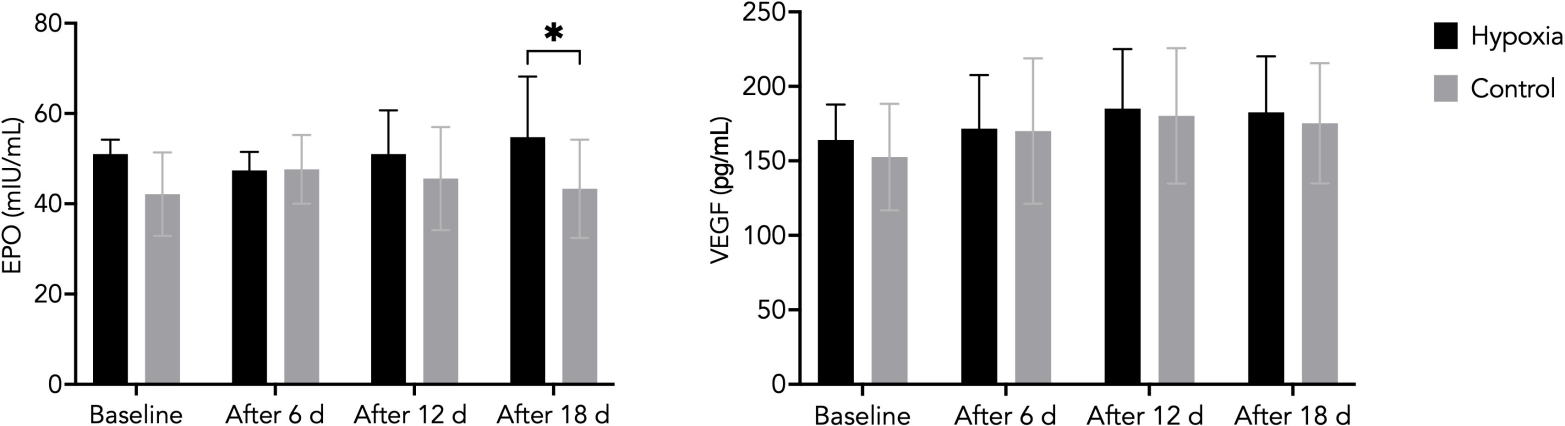
Changes in EPO and VEGF levels during the LH-TL protocol in H and C groups. Data are presented as mean ± SD. Statistically significant differences between groups: *p<0.05.

Serum erythropoietin (EPO) concentrations in the hypoxic group (H) did not change significantly compared with baseline after 6 days (47.4 ± 4.1 mIU/mL) or 12 days (51.0 ± 9.7 mIU/mL). However, after 18 days of LH-TL exposure, EPO levels were significantly higher in the H group (54.8 ± 13.4 mIU/mL) compared with the control group (C: 43.3 ± 10.9 mIU/mL; p < 0.05). No significant group × time interaction was observed (η²p = 0.09; moderate effect). Additionally, no significant main effect of time was detected, and the corresponding effect size was small (η²p = 0.017), indicating limited overall temporal changes across the study period (**Figure 2**).

VEGF concentrations remained relatively stable throughout the intervention in both groups. Slightly higher VEGF levels were observed in the H group after 6 days (171.8 ± 34.8 pg/mL) and 12 days (185.2 ± 38.6 pg/mL) compared with the C group (170.1 ± 46.1 pg/mL and 180.4 ± 42.7 pg/mL, respectively). However, neither between-group differences (η²p = 0.006; small effect) nor changes over time (η²p = 0.146; large effect) reached statistical significance (**Figure 2**).

CRP concentration increased significantly in the hypoxic group after 6 days (12.5 ± 3.0 mg/L, p < 0.001) and after 18 days (12.4 ± 2.0 mg/L, p < 0.001) compared with baseline (6.7 ± 2.1 mg/L). A significant difference between the H and C groups was also observed after 18 days of LH-TL exposure (p = 0.015).

**Figure 3:**
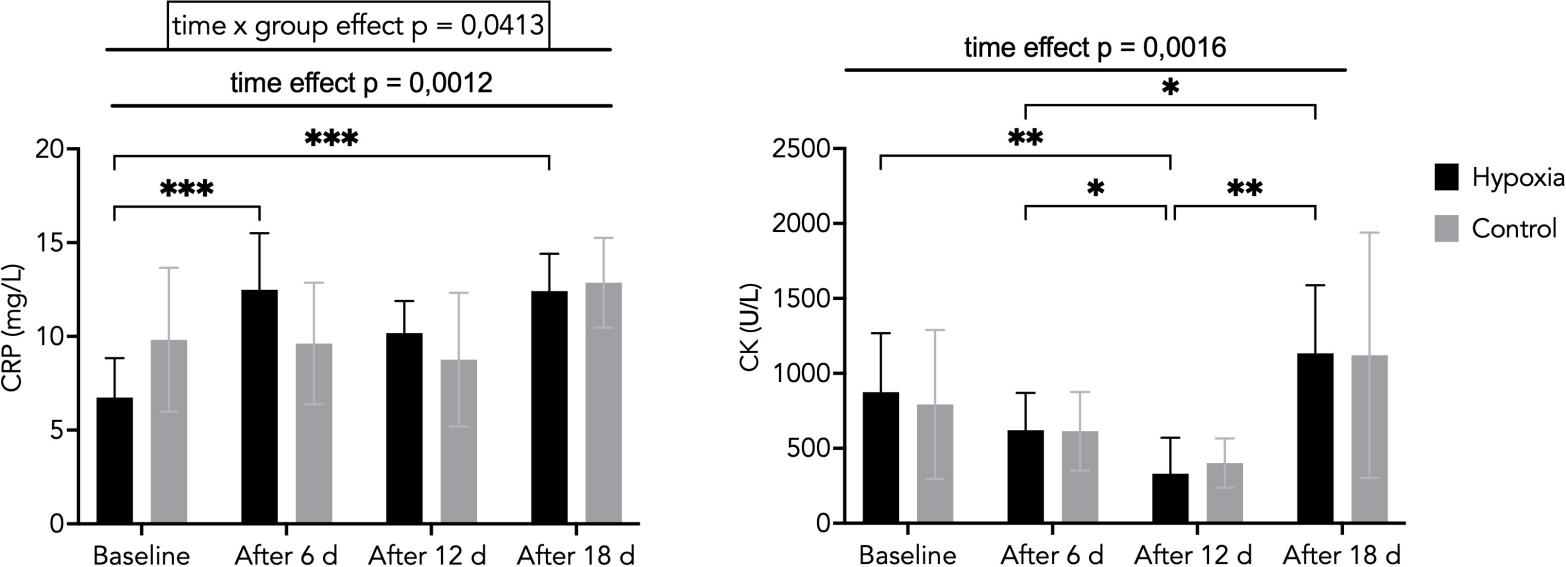
C-reactive protein (CRP) concentrations and high-sensitivity creatine kinase (CK) activity during the experiment in the H and C groups. Data are presented as mean ± SD. Statistically significant differences between groups: *p<0.05; **p<0.01, ***p<0.001.

A significant group × time interaction was detected (η²p = 0.218), along with a large main effect of time (η²p = 0.378), indicating a differential temporal response between groups (**Figure 3**). These values are consistent with an exercise- and hypoxia-induced acute-phase response rather than pathological inflammation.

CK activity varied significantly over time during the intervention. In both groups, CK levels decreased after 6 days (H: 620 ± 249 U/L; C: 614 ± 262 U/L) and 12 days (H: 331 ± 241 U/L; C: 402 ± 164 U/L) compared with baseline, followed by an increase after 18 days (H: 1133 ± 455 U/L; C: 793 ± 497 U/L).

A significant main effect of time was observed (p = 0.0016), indicating substantial temporal variation in CK activity. However, no significant interaction effect (group × time) was found (η²p = 0.008), suggesting a similar pattern of change in both groups (**Figure 3**).

**Figure 4:**
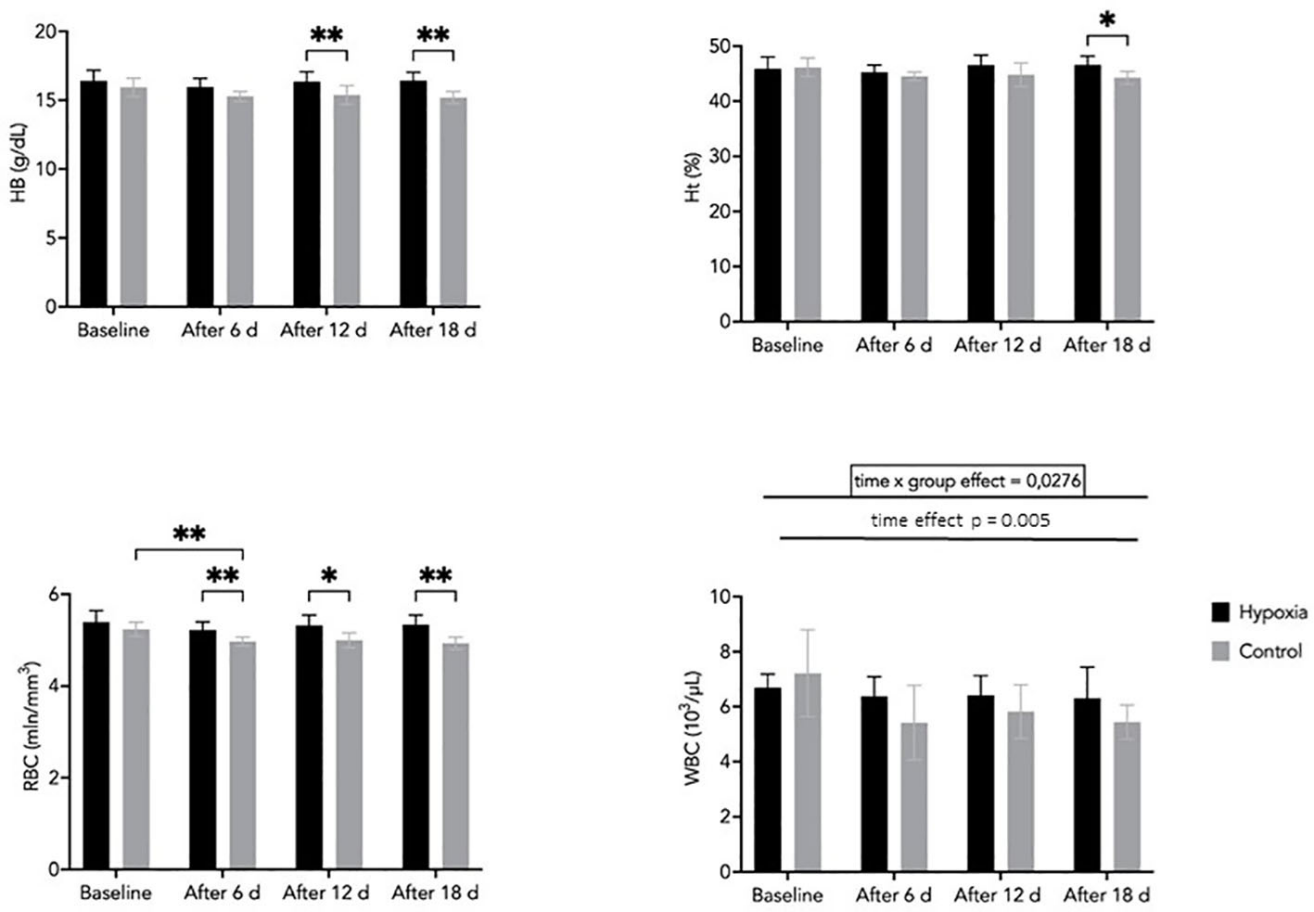
Hemoglobin (Hb), hematocrit (Ht), red blood cell (RBC), and white blood cell (WBC) concentrations during the experiment in the H and C groups. Data are presented as mean ± SD. Statistically significant differences between groups: *p<0.05; **p<0.01.

Hemoglobin concentration tended to be higher in the H group compared with the C group after 12 and 18 days; however, these differences did not reach statistical significance (p = 0.063 and p = 0.052, respectively). No significant main effects of time or group were observed. However, effect size analysis indicated a moderate effect of time (η²p = 0.119) and a large effect of group (η²p = 0.65), suggesting that variability between groups may be substantial despite the lack of consistent statistical significance (**Figure 4**).

Hematocrit values were significantly higher in the H group compared with the C group after 18 days of LH-TL exposure (p < 0.05). No consistent interaction effect (group × time) was observed, although the effect size suggested a moderate magnitude (η²p = 0.108). Similarly, the main effect of time was small to moderate (η²p = 0.074), indicating limited temporal changes during the intervention (**Figure 4**).

RBC counts remained relatively stable in the H group throughout the study, whereas a gradual decrease was observed in the C group. Between-group differences were statistically significant at days 6, 12, and 18 (p < 0.05), indicating a divergence in temporal patterns between groups. This is supported by a moderate interaction effect (group × time; η²p = 0.08). Additionally, a significant main effect of time was observed (η²p = 0.219), reflecting overall changes during the intervention (**Figure 4**).

WBC counts showed significant effects of time (η²p = 0.409) and a group × time interaction (η²p = 0.239), indicating differences in temporal patterns between groups. A slight decrease was observed in the H group from baseline (6.7 ± 0.5 ×10³/µL) to day 18 (6.3 ± 1.1 ×10³/µL), while values in the control group remained relatively stable (**Figure 4**). Although the absolute changes were small, they may reflect a subtle modulation of immune function in response to prolonged hypoxic exposure.

**Figure 5:**
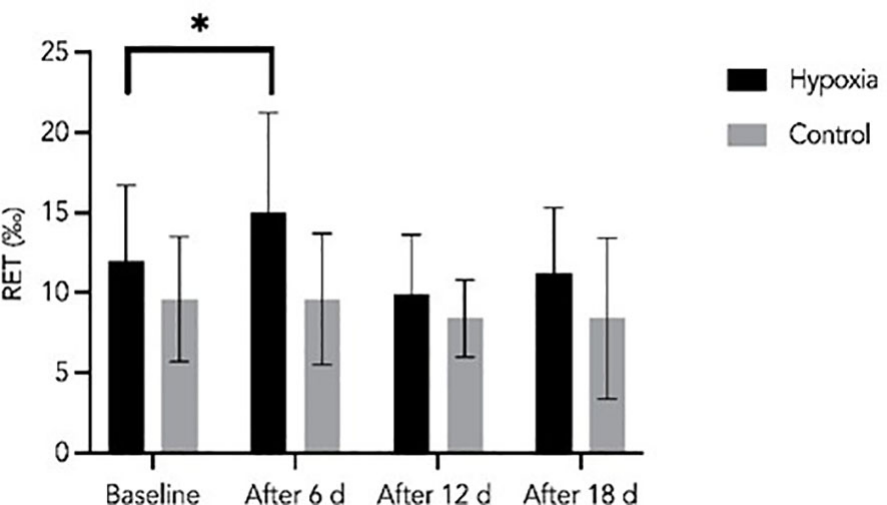
Reticulocyte levels during the experiment in the H and C groups. Data are presented as mean ± SD. Statistically significant differences between groups: *p<0.05.

Reticulocyte (Ret) levels in the H group peaked after 6 days of LH-TL exposure (15.0 ± 6.2‰) compared with baseline (12.0 ± 4.7‰), followed by a gradual decrease by day 18 (11.3 ± 4.1‰). Despite this decline, Ret levels remained higher in the H group compared with the control group throughout the intervention. A significant between-group difference was observed after 18 days (p = 0.043). Effect size analysis indicated a small interaction effect (group × time; η²p = 0.024) and a small-to-moderate effect of group (η²p = 0.067), suggesting a modest but consistent influence of hypoxic exposure on reticulocyte response (**Figure 5**).

**Figure 6:**
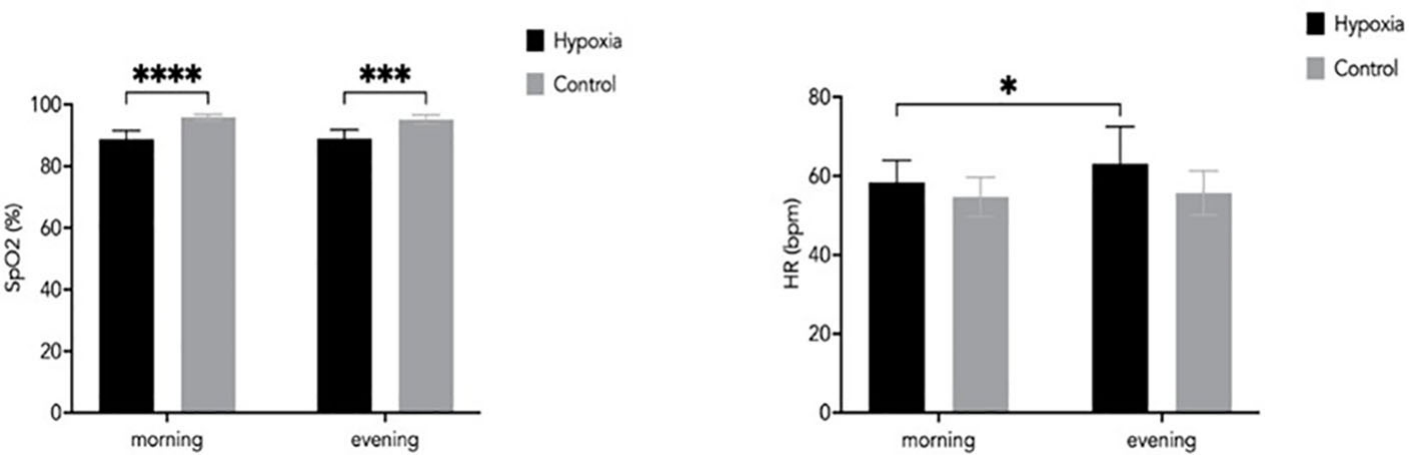
Comparison of blood oxygen saturation (SpO_2_) and heart rate (HR) between the H and C groups during morning and evening measurements. Data are presented as mean ± SD. Statistically significant differences between groups: *p<0.05; ***p<0.001; ****0.0001.

Oxygen saturation (SpO_2_) differed significantly between groups, with the H group exhibiting lower values compared with the control group both in the morning (88.88 ± 2.70% vs. 95.78 ± 0.96%, p = 0.003) and in the evening (89.14 ± 2.84% vs. 95.22 ± 1.45%, p = 0.005) (**Figure 6**).

Heart rate responses showed a different pattern. Morning HR did not differ significantly between groups (58.41 ± 5.45 bpm vs. 54.78 ± 4.78 bpm), whereas evening HR was significantly higher in the H group compared with the control group (63.16 ± 9.39 bpm vs. 55.69 ± 5.52 bpm; p = 0.022).

## Discussion

The present study examined the effects of an 18-day LH-TL intervention on hematological parameters, inflammatory markers, and hypoxia-responsive factors in elite rowers. The findings indicate that LH-TL exposure led to significant changes in erythropoietic markers and selected inflammatory indicators, with inter-individual variability.

Baseline values confirmed sufficient iron availability in both groups, reducing the likelihood that iron deficiency influenced the observed hematological responses. This is an important consideration, as iron status is a key determinant of erythropoietic efficiency during LH-TL exposure. However, the lack of randomization represents a potential source of selection bias, which may have influenced baseline characteristics and subsequent physiological responses. The 18-day duration of the LH-TL intervention allowed the assessment of both early and sustained phases of physiological adaptation, which may explain the temporal patterns observed in erythropoietic and inflammatory responses.

The increase in erythropoietic activity suggests that LH-TL effectively stimulated processes related to red blood cell production. These adaptations are consisted with hypoxia-mediated regulation of oxygen homeostasis. The magnitude of the observed changes supports the effectiveness of the applied protocol in eliciting a physiological response.

The LH-TL model is used in endurance training as it allows the combination of hypoxic exposure with the maintenance of high training intensity under normoxic conditions. The Live High-Train Low model allows athletes to effectively benefit from adaptations to low-oxygen conditions while maintaining high training intensity, which is only possible under normal (sea level) conditions (Mujika et al., 2019; Dragos et al., 2022). Although results are mixed, at least 12 hours/day over 2–3 weeks is required to induce meaningful adaptations, although responses vary considerably between individuals (Bonato et al., 2023). According to Chapman et al. (2014), a 4-week altitude camp following the LH-TL model at an altitude between 2000 and 2500 m produces an optimal acclimatization response relevant to endurance exercise at sea level. The present findings further support the concept that the effectiveness of LH-TL depends not only on hypoxic exposure itself, but also on its interaction with training load and individual responsiveness.

Erythropoiesis is considered a central mechanism underlying altitude training adaptations, primarily mediated by erythropoietin (EPO) (Saunders et al., 2019; Dragos et al., 2022). In the present study, EPO concentrations did not change significantly over time; however, higher values were observed in the hypoxic group after 18 days of LH-TL compared to the control group. This finding suggests a delayed erythropoietic response to LH-TL rather than a sustained elevation of circulating EPO. Turner et al. (2017) reported an increase in EPO level two hours after exposure to normobaric hypoxia equivalent to an altitude of ≥4200 m, following a 2-hour session. Brugniaux et al. (2006) found that an 18-day LH-TL protocol enhanced aerobic capacity and transiently increased EPO levels in elite runners, similar to our findings. Robach et al. (2006) demonstrated statistically significant increase in EPO only after exposure to 3000 m, but not at 2500 m, emphasizing the importance of altitude level. Similarly, Montero et al. (2017) presented increased concentration in EPO after 2 weeks compared to baseline. Muraoka and Gando (2012) indicated that exposing athletes to a simulated altitude of 2500 m for 10–12 hours per day leads to a significant increase in serum EPO levels the following day. Moreover, in acclimatized individuals, EPO secretion may decrease over time despite continued hypoxic exposure, reflecting a downregulation of the initial response (Hahn and Gore, 2001; Richalet et al., 1994). Therefore, the absence of prolonged EPO elevation does not necessarily indicate a lack of erythropoietic stimulation. Instead, it may reflect a physiological adjustment to sustained hypoxia, in which early signaling is followed by downstream adaptations.

In line with this interpretation, the hypoxic group demonstrated increases in RBC and reticulocyte counts over time, indicating activation of erythropoiesis. These changes suggest that erythropoietic processes may be maintained through increased sensitivity of bone marrow to EPO or through alternative hypoxia-related regulatory mechanisms (Brugniaux et al., 2006). Reticulocyte levels peaked early in the intervention and remained elevated relative to baseline, supporting the presence of a transient but functionally relevant erythropoietic response. Together, these findings indicate that LH-TL can stimulate erythropoiesis even in the absence of sustained changes in circulating EPO.

VEGF is a key regulator of angiogenesis and indirectly affects erythropoiesis by maintaining a homeostatic mechanism that preserves the capillarity and functionality of skeletal muscle during episodes of hypoxic exposure (Li et al., 2020). In the present study, VEGF levels did not show significant changes over time or between groups, although a transient increase was observed in the hypoxic group, peaking around day 12. This pattern may indicate a modest angiogenic response that did not reach statistical significance within the duration of the intervention. The VEGF levels in the hypoxic group after peaking on day 12 slightly declined by day 18, but remained elevated compared to baseline. These dynamics are in line with VEGF’s proposed role in vascular remodeling during hypoxia (Semenza, 2022). However, Wiśniewska et al. (2020) showed no significant changes in VEGF-A following LH-TL. The LH-TL protocol did not significantly affect VEGF levels, suggesting that angiogenic mechanisms may not be substantially modulated under this particular hypoxic condition, or that a longer exposure time may be required to elicit a measurable response. Therefore, the present findings suggest that angiogenic responses to short-term LH-TL may be limited at the systemic level or require a longer or more pronounced hypoxic stimulus to become detectable.

VEGF responses may also be influenced by factors beyond hypoxia alone, including training status and cumulative training load. In endurance-trained individuals, angiogenic signaling is often more tightly regulated, and baseline capillarization may already be elevated, which can attenuate further increases in circulating VEGF in response to additional stimuli (Hoier and Hellsten, 2014). Therefore, the lack of significant changes in VEGF observed in the present study may reflect an already well-adapted vascular system in elite athletes. Moreover, it should be noted that circulating VEGF may not fully reflect local, muscle-level angiogenic processes, which are considered more sensitive indicators of vascular adaptation to hypoxia and exercise (Lundby and Robach, 2016; Semenza, 2012). The present study was designed to assess systemic physiological responses in an applied elite sport setting and did not include the evaluation of intracellular signaling pathways, such as hypoxia-inducible factors HIFs. Accordingly, downstream circulating markers, including EPO and VEGF, were used as indirect indicators of hypoxia-related adaptation, consistent with previous studies in altitude training research (Chapman et al., 2010; Brugniaux et al., 2006; Robach et al., 2006).

Hypoxic exposure may modulate inflammatory signaling pathways through interactions between oxygen-sensitive regulatory pathways and immune signaling. In the present study, CRP levels increased during the intervention, whereas CK did not show a corresponding elevation. A similar dissociation between CRP and CK has been reported in previous studies, where inflammatory activation occurred without clear evidence of muscle damage (Khalafi et al., 2023). Temporal changes in inflammatory markers observed in this study are consistent with earlier findings. Hohenauer et al. (2024) observed elevated CRP levels 24 hours after hypoxic exposure, followed by a subsequent decrease at 48 and 72 hours, indicating a transient inflammatory response. Moreover, the authors demonstrated a significant time effect and a peak in CK activity after 24 hours of hypoxic exposure (Hohenauer et al., 2024). Similarly, Turner et al. (2017) reported that CRP levels peaked after 5 and 18 days of the LH-TL method, accompanied by variable CK responses over time. These patterns support the interpretation that hypoxia-induced inflammation is dynamic and closely linked to training load.

Although CRP values in the present study exceeded 10 mg/L; however, CRP is a non-specific marker of systemic inflammation, and its interpretation should be considered within the physiological context of elite athletes. Elevated CRP levels in this population are commonly associated with accumulated training stress and recovery processes rather than pathological inflammation. Therefore, the increase observed during the LH-TL protocol most likely reflects a transient response to inflammatory combined hypoxic exposure and exercise load, potentially mediated by hypoxia-related signaling pathways (Hohenauer et al., 2024; Baygutalp et al., 2021; Li et al., 2020; Pham et al., 2021).

This interpretation is further supported by the lack of a concomitant increase in CK, suggesting that the inflammatory response was not primarily driven by muscle damage (Hohenauer et al., 2024; Baygutalp et al., 2021; Li et al., 2020; Pham et al., 2021). The decrease in CK observed during the intervention may additionally indicate progressive adaptation to training load. Despite the increase over time in the hypoxic group, between-group differences in CRP did not reach statistical significance, indicating a moderate and transient inflammatory response.

Taken together, these findings suggest that the inflammatory response observed during LH-TL represents a physiological and adaptive process rather than a maladaptive or pathological state.

Hematological adaptations represent a primary target of altitude training. In the present study, most hematological variables remained relatively stable over time, with only modest differences observed between groups rather than pronounced time-dependent increases. This pattern is consistent with previous reports indicating that the magnitude of hematological responses depends strongly on the hypoxic dose and exposure duration. For example, Robach et al. (2006) reported greater increases in hemoglobin and hematocrit after exposure to 3000 m compared to 2500 m. In contrast, Hauser et al. (2017) and Wahl et al. (2013) observed increases in Hb mass and RBC count following LH-TL interventions in endurance athletes. However, more recent evidence highlights substantial inter-individual variability in hematological responses to hypoxic training (Bonato et al., 2023; Chen et al., 2023). Therefore, the relatively moderate changes observed in the present study may reflect the applied hypoxic dose, intervention duration, or individual responsiveness of the athletes (Chen et al., 2023; Wilber et al., 2007). Similar findings have also been reported by Zubieta-Calleja et al. (2007), where, following short-term altitude exposure, hematocrit increased within the first week.

A slight but consistent decrease in WBC observed in the hypoxic group, accompanied by a significant group × time interaction, suggests a differential temporal response between groups and a subtle modulation of immune function in response to prolonged hypoxic exposure. Given that all values remained within physiological ranges, this response is more likely to reflect redistribution of leukocytes rather than true immunosuppression. Such mechanisms have been described in both endurance training and hypoxic conditions, where immune cells transiently shift between the circulation and peripheral tissues (Walsh et al., 2011; Simpson et al., 2020; Hartmann et al., 2000). These alterations may be further influenced by hypoxia-related activation of stress pathways and inflammatory pathways (Hartmann et al., 2000; Mazzeo, 2008), particularly under combined training load and environmental stress during a training camp. Taken together, these findings suggest that the LH-TL method induces a coordinated physiological response involving both inflammatory activation and modulation of immune response (Mazzeo, 2008; Lundby and Robach, 2016).

In the hypoxic group, reticulocyte levels peaked early, on day 6, in the hypoxic group and subsequently declined while remaining above baseline values. Although reticulocyte counts decreased after the initial peak, they remained above baseline values, indicating sustained stimulation of erythropoiesis. This pattern is consistent with the typical time course of erythropoietic responses to hypoxia, which occur within the first days of exposure as bone marrow activity increases (Turner et al., 2017; Montero et al., 2017). Previous LH-TL studies have also reported increases in reticulocyte levels during hypoxic training interventions. For example, Chapman et al. (2019) observed elevated reticulocyte counts after three weeks of altitude exposure at approximately 1600 m. Similarly, Muraoka and Gando (2012) reported significant increases in reticulocyte levels after 3–4 weeks of LH-TL exposure (10–12 h/day at ~2500 m). However, other studies have reported variable responses, with Ashenden et al. (2000) and Robach et al. (2006) showing minimal or no changes in Ret counts during shorter interventions, supporting the notion that both the magnitude and timing of this response depend on hypoxic dose and individual variability (Chen et al., 2023; Bonato et al., 2023). Together, these findings suggest that short-term LH-TL may primarily induce early and transient erythropoietic activation rather than large increases in mature erythrocyte indices.

Changes in oxygen saturation and heart rate observed during the LH-TL protocol reflect the expected physiological response to hypoxic exposure. The hypoxic group demonstrated reduced SpO_2_ levels compared to the control group, consistent with previous studies reporting decreased oxygen saturation under normobaric hypoxia. Similarly, Hohenauer et al. (2024) reported reduced SpO_2_ levels in the group exposed to hypoxia compared to a control group. Chinapong et al. (2021) also observed a decrease in SpO_2_ in the hypoxic group, reaching levels between 82-89%. According to Mourot (2018), SpO_2_ decreases gradually with increasing altitude, while Furian et al. (2022) confirmed that SpO_2_ levels decline with increasing degrees of hypoxia.

Heart rate responses further support the presence of hypoxia-induced physiological stress. In the hypoxic group, evening heart rate values were higher than morning values, which may reflect elevated sympathetic nervous system activity and cumulative daily load. In contrast, no significant changes in SpO_2_ or HR were observed in the control group, confirming stable physiological conditions under normoxia. It should be noted that heart rate was analyzed as a complementary indicator of physiological stress, and baseline-adjusted or time-point-specific analyses were not performed due to the exploratory nature of this variable and the limited sample size.

In conclusion, the study demonstrates that an 18-day LH-TL intervention led to an overall increase in oxygen-carrying capacity and transient inflammatory responses, supporting its role in strategies aimed at enhancing endurance adaptations in elite athletes. Although erythropoietin responses were not sustained over time, the overall hematological profile indicates activation of erythropoiesis and a potential improvement in oxygen transport capacity. The observed increase in CRP, in the absence of elevated CK, suggests a transient and adaptive inflammatory response rather than muscle damage. These findings highlight the integrative nature of physiological adaptations to LH-TL, involving interactions between erythropoietic regulation, inflammatory signaling, and vascular responses. Importantly, the results support the use of LH-TL as an effective strategy in endurance training, while also emphasizing the role of individual variability in determining the magnitude of adaptation.

The present study is limited by relatively low statistical power (1-β ≈ 0.10), primarily due to the small sample size, particularly in the control group. As a result, the study may not have been sufficiently sensitive to detect small-to-moderate effects. However, several variables demonstrated moderate to large effect sizes despite non-significant p-values, suggesting that meaningful physiological adaptations may have occurred. This limitation is common in studies involving elite athletes, where participant availability is restricted but experimental control is high. Future studies with larger cohorts are needed to confirm and extend these findings.

## Summary

This study investigated the effects of an 18-day Live High-Train Low (LH-TL) protocol on erythropoietin (EPO), vascular endothelial growth factor (VEGF), inflammatory markers, and hematological parameters in elite rowers. The intervention induced measurable hematological and inflammatory responses without evidence of excessive muscle damage. Notably, the increase in C-reactive protein (CRP) in the absence of related elevations in creatine kinase (CK) suggests a systemic inflammatory response rather than muscle injury.

An increase in evening heart rate was also observed, likely reflecting cumulative physiological strain associated with combined hypoxic exposure and training load. Hematological responses, including elevated reticulocyte counts with relatively stable red blood cell indices, indicate activation of erythropoiesis during the intervention. These adaptations may contribute to improved oxygen transport capacity and support endurance performance.

The study is limited by a small sample size (n = 13, including n = 5 in the control group), resulting in low statistical power and reduced sensitivity to detect moderate effects. Consequently, the risk of type II error is increased, and the findings should be interpreted with caution. Despite this limitation, selected responses, such as changes in CRP and reticulocyte levels, may still be physiologically meaningful in elite athletes, highlighting the importance of considering practical relevance alongside statistical significance. Future research should focus on long-term physiological adaptations, performance outcomes, and recovery kinetics following LH-TL interventions. Expanding the range of biomarkers to include indicators of oxidative stress and immune regulation may provide further insight into underlying mechanisms. Additionally, addressing inter-individual variability in response to hypoxia may support the development of more individualized training strategies. A limitation of the present study is that the perfusion index was not assessed during SpO_2_ measurements.

From a practical perspective, LH-TL appear to stimulate erythropoietic activity while inducing a moderate and transient inflammatory response. These findings emphasize the importance of individualized management of hypoxic dose, including altitude level, exposure duration, and integration with training load. Given the variability in physiological responses, regular monitoring of hematological and inflammatory markers may support optimization of adaptation and recovery.

Overall, defining an optimal hypoxic dose remains challenging, particularly in elite athletes, and likely depends on individual responsiveness, training phase, and competition schedule. A personalized approach, supported by ongoing physiological monitoring, may therefore be more effective than standardized protocols.

## Data Availability

The raw data supporting the conclusions of this article will be made available by the authors, without undue reservation.
